# Testing a Threshold-Based Bed Bug Management Approach in Apartment Buildings

**DOI:** 10.3390/insects8030076

**Published:** 2017-07-26

**Authors:** Narinderpal Singh, Changlu Wang, Chen Zha, Richard Cooper, Mark Robson

**Affiliations:** 1 Department of Entomology, Rutgers-The State University of New Jersey, New Brunswick, NJ 08901, USA; singh.narinderpal@gmail.com (N.S.); cz166@scarletmail.rutgers.edu (C.Z.); rick.cooper@cooperpest.com (R.C.); 2 Department of Plant Biology, Rutgers-The State University of New Jersey, New Brunswick, NJ 08901, USA; robson@aesop.rutgers.edu

**Keywords:** *Cimex lectularius*, pest management, multi-unit dwelling

## Abstract

We tested a threshold-based bed bug (*Cimex lectularius* L.) management approach with the goal of achieving elimination with minimal or no insecticide application. Thirty-two bed bug infested apartments were identified. These apartments were divided into four treatment groups based on apartment size and initial bed bug count, obtained through a combination of visual inspection and bed bug monitors: I- Non-chemical only in apartments with 1–12 bed bug count, II- Chemical control only in apartments with 1–12 bed bug count, III- Non-chemical and chemical control in apartments with >12 bed bug count, and IV- Chemical control only in apartments with ≥11 bed bug count. All apartments were monitored or treated once every two weeks for a maximum of 28 wk. Treatment I eliminated bed bugs in a similar amount of time to treatment II. Time to eliminate bed bugs was similar between treatment III and IV but required significantly less insecticide spray in treatment III than that in treatment IV. A threshold-based management approach (non-chemical only or non-chemical and chemical) can eliminate bed bugs in a similar amount of time, using little to no pesticide compared to a chemical only approach.

## 1. Introduction

The common bed bug, *Cimex lectularius* L. (Hemiptera: Cimicidae), once thought to be largely eradicated in the U.S., has reemerged in recent years, particularly in urban centers. In 2016, revenue from professional bed bug control services and products in U.S. reached US $611.2 million [1]. Nationwide, 85% of the pest control companies charged >US $500 for treating each bed bug infestation [2,3]. In addition to the cost for treatment of infestations, people often throw away their infested furniture and personal belongings in an attempt to eliminate bed bugs. The economic loss for hotel owners, furniture rental companies, and rental home owners is often much higher due to loss of business and lawsuits [4]. Low-income communities suffer disproportionally higher bed bug infestation rates [5,6,7,8]. Numerous insecticide products are available for the treatment of bed bugs and are almost always included in bed bug control programs regardless of infestation levels [9,10,11]. Such reliance upon pesticides is of concern given that bed bugs have developed resistance to most of the insecticide classes used for bed bug control today [12]. High levels of pyrethroid resistance in bed bugs has been documented in many field-collected strains [13]. In addition to mutations related to target site (voltage-gated sodium channel) insensitivity [14], bed bugs have also evolved multiple other resistance mechanisms [15,16]. Application of insecticides on or around sleeping or resting furniture also causes concern of human exposure to insecticides.

Detailed descriptions of bed bug inspection, prevention, and individual control techniques and tools are available [17,18]. Bed bugs are difficult to control because of their secretive behavior, the lack of effective residual insecticides, and insecticide resistance. Recent research found non-chemical tools and methods such as frequent laundering, decluttering, mattress encasements, steam treatments, heat chambers, and whole-house heat treatment to be valuable in the management of bed bug infestations [11,19,20,21]. Using bed bug interceptors is much more reliable than using trained dogs, visual inspections, or resident reports for detecting new infestations and evaluating control effectiveness [21,22]. Post-treatment monitoring and re-treatment is necessary to ensure bed bug elimination. Interception devices installed under furniture legs are cost-effective monitoring tools and help reduce bed bug numbers [23,24,25]. The published research demonstrates that integrated pest management (IPM) is the best approach for successful suppression of bed bug infestations [26]. For example, Cooper et al. (2015) evaluated an IPM program in a low-income housing community over a 12 month period [27]. The program included education of residents and staff, and implementation of a combination of monitors, steam, encasement, decluttering, laundering, and limited insecticide applications. Results show that the number of treatment visits required to eliminate an infestation is correlated with infestation levels, and moderate to severe infestations can be eliminated without using large amounts of pesticides. Although past IPM studies demonstrated reduction of bed bug populations of up to 98% [20,28,29,30], there are no guidelines on how to decide which methods and procedures should be used based on infestation levels to safely and economically eliminate bed bug infestations.

The indiscriminate use of insecticides by homeowners to control bed bugs is a big concern [31,32]. Some pest control companies rely solely on the use of insecticides to treat bed bug infestations regardless of the infestation levels. Also most professional pest control companies require residents to prepare their residence following an extensive preparation list, regardless of infestation level, often refusing to treat if preparations have not been carried out in full [17]. The preparations are time-consuming, labor-intensive and often over-burdensome, especially for individuals that are elderly or handicapped. Moreover, these preparations dislocate bed bugs to un-infested areas in the home. The objective of this study was to evaluate a threshold-based management approach (based upon infestation level), with limited resident preparation. Our specific questions were: (1) whether non-chemical plus chemical treatment will result in faster bed bug eradication and reduced insecticide use compared to chemical control alone for moderate to high-level bed bug infestations; and (2) whether non-chemical control alone can eliminate low-level infestations at similar speed as chemical control alone.

## 2. Materials and Methods

### 2.1. Study Site

The study was conducted in Irvington, New Jersey between May and December, 2014, in two 11-story high-rise apartment buildings consisting of a total of 409 apartments occupied by seniors (>62 yr old) or disabled residents. These two buildings had bed bug (*C. lectularius*) infestations for at least 5 yr prior to this study. In an effort to combat bed bugs in their apartments, residents used various methods including insecticide sprays, diatomaceous earth dust, discarding infested furniture and other personal belongings, and installing encasements to the mattresses and box springs. A housing staff member helped residents by applying steam to the infested furniture using a steam machine (The Steamax, Amerivap Systems, Dawsonville, GA, USA). The researchers from Rutgers University treated approximately 60 apartments using both chemical and non-chemical methods 1–2 yr prior to this study.

### 2.2. Selection of Apartments 

We first conducted building-wide inspections to identify bed bug infested apartments. Climbup Insect Interceptors (Susan McKnight, Inc., Memphis, TN, USA), hereafter referred to as interceptors, were installed under the legs of beds and upholstered furniture and were inspected after 12–15 d placement. An average of 9 interceptors (range: 6–14) were placed per apartment. Total bed bug count from the interceptors laid per apartment was adjusted by dividing the counts by number of days of placement then multiplied by 14 to yield 14 d interceptor counts. Immediately after inspecting the interceptors, a thorough visual inspection was conducted in apartments with bed bugs or with suspected infestations based on previous infestation history or resident report. The visual inspection was helpful for providing an accurate estimation of bed bug population levels in those that had been detected by interceptors, and to identify additional bed bug infested units that had not been identified by the interceptors. The total bed bug count per apartment was calculated by adding the 14 d interceptor count and the visual inspection count. All bed bug counts mentioned in the paper were based on the sum of the 14 d interceptor count and the visual inspection count unless indicated otherwise. A total of 83 bed bug (*C. lectularius*) infested apartments (20% of the total occupied units) were identified. 

We initially selected 37 apartments (35 from the first building and 2 from the second building). Selection was based on (1) residents agreeing to participate in the study, and (2) the apartment not being excessively cluttered. Five apartments were dropped from the study over the course of the study. Four of them were due to residents’ refusal to continue the biweekly (every two weeks) inspections or lack of access during many of the follow-up visits. One of them was due to incorrect treatment as defined by the treatment protocol.

**Figure 1:**
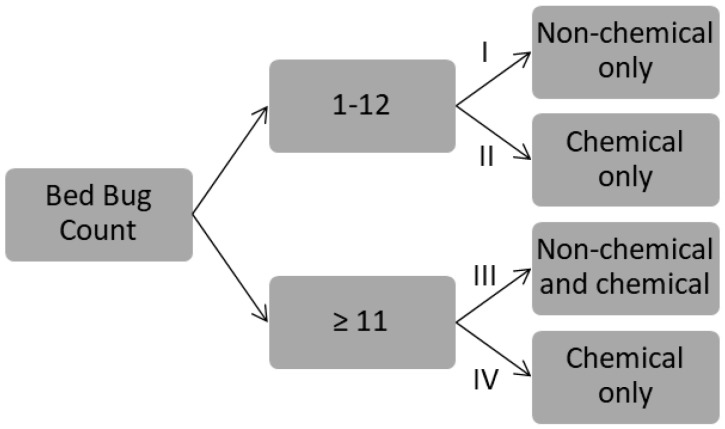
Diagram showing the treatment groups based on initial bed bug count.

The 32 selected apartments were evenly divided into four treatment groups based on apartment size and infestation levels: (I) Non-chemical control only in apartments with low-level infestation (1–12 bed bug counts); (II) Chemical control only in apartments with low-level infestation (1–12 bed bug counts); (III) Non-chemical and chemical control in apartments with medium or high-level infestation (>12 bed bug counts); and (IV) Chemical control only in apartments with medium or high-level infestation (≥11 bed bug counts) (Figure 1). Treatment IV had two apartments with counts (11 and 12) overlapping with the bed bug count range in I and II groups. It would have been more appropriate if the apartment with the 11 bed bug count had been assigned to I or II group. However, we kept this apartment in the data analysis due to the small sample size. A bed bug educational seminar was delivered to building residents, along with bed bug fact sheets to increase their knowledge about bed bug prevention and control. The fact sheet included (a) bed bug identification, symptoms of bed bug bites and their impact on human health; (b) bed bug biology and behavior, typical signs of bed bug infestation; (c) how to prevent introducing new bed bugs; and (d) how to control existing infestations by both chemical and non-chemical methods. In addition, we handed out bed bug fact sheets to each resident who enrolled in this study during visits to their apartment. Education of clients using printed materials or by face-to-face conversation was typical practice among pest control companies in the U.S. Therefore, we did not try to exclude the II and IV treatment groups in our education effort.

### 2.3. Treatment Methods

Within three weeks following the initial inspection of the apartments, residents from the 32 selected apartments were asked to stop using any insecticides. We did not ask residents to do extensive preparations typical of those required by most pest control companies, such as removing items around the beds and sofas, stripping bed linens, removing mattresses and box springs, or emptying closets and drawers. Instead, we only asked residents to reduce clutter (or place clutter in sealed plastic bags) and launder their bed linens frequently after our inspection. In treatment I, the following non-chemical strategies were used during initial treatment and follow-up visits:

- beds, sofas, upholstered furniture, and their surrounding areas were inspected and bed bugs were hand-removed with forceps or killed using a “Steamax” steam machine;
- bed bug-proof mattress encasements (Protect-A-Bed, Wheeling, IL, USA) were installed during initial treatment in all apartments;
- bed linens and pillow covers were bagged in plastic bags for washing by residents
- clutter under or surrounding the beds and sofas was removed or reduced;
- interceptors were installed under legs of beds and upholstered furniture after initial treatment, and then were inspected/cleaned during biweekly follow-up visits; and
- visual inspection of beds and upholstered furniture was conducted during each biweekly visit, and any visible bed bugs were hand-removed or killed using a “Steamax” steam machine.

Treatment III was same as treatment I with the addition of the following chemical control methods:

- Transport GHP (0.11% a.i. diluted solution which contains acetamiprid and bifenthrin at 0.83:1 ratio; FMC Corporation, Philadelphia, PA, USA) spray was applied with a one-gallon B&G sprayer (Univar, Edison, NJ, USA) on bed frames, headboards, footboards, the underside of furniture, as well as around the legs, along baseboards, and along the base of the wall; and
- Cynoff dust (0.075% zeta-cypermethrin, 0.15% piperonyl butoxide; FMC Corporation, Philadelphia, PA, USA) was applied using a bulb duster to electrical outlets and switch plates only during initial treatment.

These insecticides are effective in controlling bed bugs [33,34,35]. Application rates of both insecticides were based on label directions. In treatments II and IV, chemical control methods were used in the same manner as in treatment III. Non-chemical intervention was not applied, except that interceptors were installed under legs of beds and upholstered furniture for 14 d every 4 wk to obtain bed bug counts. The interceptors were inspected after 14 d and then removed for the next 4 wk to minimize the effect of interceptors on population reduction [25]. Visual inspection of beds and upholstered furniture was conducted during each biweekly visit. All treatment tasks were carried out by Rutgers University researchers. Two or three researchers serviced each apartment during each visit. The labor (time in apartment multiplied by number of researchers) spent in each serviced apartment was recorded during each visit.

Follow-up visits continued every two weeks until 24 wk or the following criteria were met simultaneously over three consecutive visits: (1) no new reports of bed bug activity or bite symptoms by the resident; and (2) no bed bug activity observed in interceptors and a visual inspection of beds and upholstered furniture in treatments I and III, or (3) no bed bug activity observed in three consecutive visual inspections of beds and upholstered furniture and at least one inspection without bed bug activity in interceptors in treatments II and IV. Spot treatment (i.e. applying insecticide directly to live bed bugs or to harborages where bed bug presence was suspected) using Transport GHP was conducted if bed bugs were detected in treatments II and IV. Steam treatment was applied if bed bugs were detected in treatments I and III. In addition to steam treatment, Transport GHP was applied if bed bug counts were ≥12 in treatment III. Interviews of residents were conducted before the initial treatment and at the end of the study to obtain information on residents’ pesticide use and laundering practice. This study received Rutgers University Institutional Review Board approval (protocol number E14-097). 

### 2.4. Statistical Analysis

One-way analysis of variance (ANOVA) was used to compare mean bed bug count (sum of 14 interceptor count and visual inspection count), insecticide usage, initial and total service time until bed bug elimination. Bed bug counts and total amount of spray were logarithmically transformed; insecticide dust use was square root transformed before ANOVA. Tukey’s HSD test (*p* = 0.05) was used to separate the means. Logarithmically transformed bed bug count reduction data over the observation period were analyzed using mixed model (Proc MIX in SAS software). Kruskal-Wallis test was used to compare the median number of re-treatments before bed bugs were eliminated and median number of visits before eliminating an infestation. All analyses were conducted using SAS software 9.3 [36]. 

## 3. Results

Among the 32 apartments selected for this study, the initial median (min, max) bed bug count based on interceptors only was 5 (0, 42) and the median (min, max) bed bug count based on visual inspection only was 3 (0, 200). The initial bed bug count based on a combination of interceptors and a visual inspection was summarized in Table 1. There were significant differences in the mean bed bug count among the four treatment groups (F = 16.1; df = 3, 28; *p* < 0.001). However, the mean bed bug counts between I and II (low population level treatments) or between III and IV (medium or high population level treatments) were not significantly different (Tukey’s HSD test, *p* > 0.05). One apartment from treatment I was not inspected after 10 wk due to refusal of access from the resident. This apartment had only one male adult bed bug during 6 wk inspection and one small nymph during 10 wk inspection, and no bed bugs were found during the 4 and 6 wk inspections. Thus, we can reasonably assume that this apartment no longer had bed bugs at 28 wk. The elimination rate in treatments I to IV was 100, 86, 89, and 88%, respectively, at 28 wk. The maximum bed bug count among the apartments that still had bed bugs was 9. 

The treatment information is shown in Table 2. During the initial treatment, the mean amount of Cynoff dust applied per apartment was similar (F = 0.34; df = 2, 21; *p* = 0.72) in the three groups treated with pesticides (II, III and IV). However, the total diluted insecticide spray used per apartment in the three treatments were significantly different (F = 3.6; df = 2, 21; *p* = 0.04), with significantly less (58%) spray being used in treatment III than in treatment IV (Tukey’s HSD test, *p* < 0.05). The mean initial treatment time per apartment was not significantly different among the treatments (F = 1.6; df = 3, 28; *p* = 0.22). Excluding the four apartments that were never eliminated or terminated early, the labor (mean ± SEM) required per apartment until bed bug elimination was 112 ± 11, 197 ± 30, 295 ± 43, 350 ± 65 minutes, respectively. They are significantly different (F = 6.4, df = 3, 24; *p* = 0.003) with treatment I requiring significantly less labor time than III and IV (Tukey’s HSD test, *p* < 0.05,). There was no significant difference in the median number of re-treatments between I and II (χ^2^ = 2.6; df = 1; *p* = 0.10) or between III and IV (χ^2^ = 1.6; df = 1; *p* = 0.20). Similarly, there was no significant difference in the median number of biweekly visits per apartment between I and II (χ^2^ = 2.1; df = 1; *p* = 0.15) or between III and IV (χ^2^ = 0.3; df = 1; *p* = 0.61) before bed bugs were eliminated. The number of biweekly visits per apartment is larger than the number of re-treatments because no treatments were done if bed bugs were not detected.

**Figure 2:**
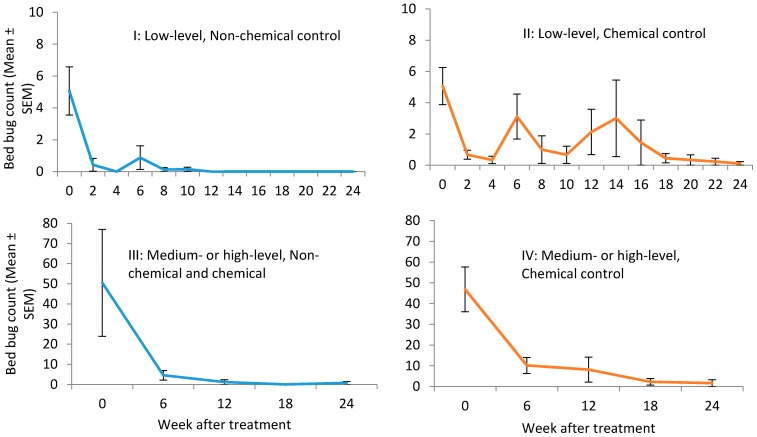
Changes in bed bug count after treatment.

Changes in bed bug count after treatment are shown in Figure 2. Bed bug count reduction in treatments I and II were not analyzed because the initial counts were very low. Bed bug count reduction data in treatments III and IV were only compared at 6, 12, 18, and 24 wk post-treatment because interceptors were not placed in treatment IV during other observation periods. The mean bed bug count reduction in treatment III at 6, 12, 18, and 24 wk was 75 ± 18, 91 ± 8, 100 ± 0, 95 ± 5%, respectively. The mean bed bug count reduction in treatment IV at 6, 12, 18, and 24 wk was 74 ± 9, 85 ± 8, 96 ± 2, 98 ± 2%, respectively. There was no significant difference in the bed bug reduction between treatment III and IV during each of the observation periods, but there were significant differences between the observation periods (F = 4.4; df = 7, 36; *p* = 0.001). 

## 4. Discussion

A primary goal of this study was to develop guidelines for what treatment strategies should be used to eliminate bed bug infestations effectively and economically, while minimizing the application of pesticides. We divided bed bug infestations into two density levels based on a combination of interceptor counts and visual inspection counts. At low bed bug population levels (≤12 bed bugs), bed bugs were eliminated using non-chemical methods alone (treatment I) as efficiently as those treated with pesticides alone (treatment II). At higher population levels (≥11 bed bugs), a combination of non-chemical and chemical treatment achieved similar speed of elimination and required 58% less insecticide spray compared to the chemical only treatment. Pest management professionals (PMPs) could use similar thresholds to determine their treatment strategies in apartment buildings. During a building-wide bed bug management study, this threshold-based treatment principle was applied to four apartments where the total bed bug count based on interceptor counts and visual inspection was ≤5. Bed bugs were eliminated from all four apartments after one treatment using non-chemical methods [27]. In another study, low-level bed bug infestations (bed bug count ≤10 based on interceptor count and visual inspection) were eliminated in 50% of apartments after 16 wk by mass trapping alone [25]. Wang et al. (2012) compared three bed bug treatment strategies in apartments [28]. Among the apartments with <10 bed bugs based on interceptor count, bed bugs in 5 of the 6 apartments treated with non-chemical methods alone were eliminated after 10 wk. Together, these studies support the use of a threshold-based approach to avoid or minimize insecticide applications. A large-scale survey of bed bug infestations in apartment buildings revealed 64% of the infestations had ≤10 bed bugs based on interceptor counts only [6]. Although a different sampling method than that in the current study was used, the number still indicates that high portion of the natural bed bug infestations fall into the low-level infestation category and could be eliminated with little to no insecticide application. In our study, heavily cluttered apartments were excluded from the study. Thus, while our results apply to the majority of infested apartments, further investigation is required to determine if they can also be applied to bed bug infested apartments with excessive clutter. 

Professional pest control companies typically require home preparations before treatments. In a survey of the pest management industry in 2015, 71% of pest management companies indicated they require full preparation prior to treatment [3]. The preparation list often includes lifting mattresses and box springs, emptying and bagging the contents of closets and drawers, removing items near beds and upholstered furniture. It requires an average of 15 man hours per apartment, and can cost up to $200 per apartment to complete preparations. Pest management professionals often refuse to treat apartments that are not fully prepared. These preparations are expensive [17] and over-burdensome, particularly for elderly or disabled residents [2,7]. Additionally, extensive preparation prior to treatment can disrupt and result in the relocation of bed bugs preventing proper evaluation of the infestation and making control more difficult [17]. Residents usually do not know where bed bugs are hiding and may relocate the bed bugs along with the infested items during preparation. In our study, residents were not asked to do extensive preparations prior to each treatment visit and most infestations were eliminated at similar speed as in Cooper et al. (2015b) [27]. We inspected clothes, bed sheets, or any other clutter surrounding the beds before we ask the tenants to bag them. Therefore, PMPs should adopt client-specific preparations when conducting bed bug control, rather than using a one-size-fits-all protocol for all apartments regardless of infestation size or apartment living conditions. Only a small portion of the clients may require significant preparation (furniture removal, declutter, etc.). In this study, very cluttered infested apartments were excluded from the study to keep homogeneity of the test apartments. In the future it would be interesting to compare a limited-preparation protocol to full preparation in apartments that are excessively cluttered. When preparation is needed, it would be best done by PMPs who are familiar with the bed bug biology and would be able to treat the infested items immediately during preparation.

The lack of significant difference in the initial treatment time among the four treatment groups was probably a result of all apartments being carefully inspected immediately before the non-chemical and/or chemical treatment. The inspection was necessary for identifying the harborages and determining where to apply treatments. If only counting the intervention time, then there could be significant differences among the treatments. The median number of visits required for eliminating low-level infestations (treatments I and II) was similar to that based on pest control industry survey [3]. The similar number of re-treatments required between treatment I and II shows when bed bug numbers are low, either non-chemical only or chemical only treatment method can easily eliminate the bed bugs. A much greater (but not statistically different) difference in the number of re-treatments (median number of 2 versus 6) was found between treatment III and IV. More studies are needed to determine whether a combination of non-chemical and chemical treatment would significantly reduce the need for re-treatment compared to chemical only treatment for eliminating moderate and high-level infestations. 

The comparative costs of the four treatments were not calculated considering that the materials and labor cost vary greatly depending on the brands, quality, geographical region, etc. However, we summarized the median amount of insecticide use, the service time needed before elimination, and the number of service visits before elimination. Technicians, housing staff, and residents can easily estimate the cost based on the products they choose and prevailing labor rate for bed bug control service. In treatments I and II, the major material cost is zippered fabric mattress encasements, which costs $25–90 for a queen size based on internet vendor (https://www.amazon.com). But a zippered plastic encasement only costs $6–10, which also serves the purpose of protecting the bed. A one- bedroom apartment typically requires two encasements. Labor accounted for the majority of the bed bug management cost assuming labor rate being $50 per hour. The cost of client education and the travel time to the bed bug site were not included. Compared to chemical only treatment, the non-chemical or combination of non-chemical and chemical treatment required similar amount of time. Therefore, the non-chemical or non-chemical plus chemical management approach would not significantly increase the cost compared with chemical control but will have the advantage of no or limited insecticide usage. 

We observed the following factors that contributed to the failure or slow speed of bed bug elimination in some apartments during the study period: (1) residents refused biweekly treatment visits in spite of bed bugs still being detected in low numbers, (2) sleeping area was very cluttered, (3) residents rarely washed their bed linen that had bed bug signs, and (4) residents sleeping on sofa or hard-to-treat wooden furniture. A resident from treatment I refused inspections after 10 wk in spite of the fact that we found one male bed bug at 8 wk and one small nymph at 10 wk. No bed bugs were found at wk 4 and 6 wk. This apartment used to have high numbers of bed bugs and the resident was bothered by bed bugs prior to our study, but was not concerned about the presence of very low number of bed bugs identified during our inspections. One apartment in treatment II had a heavy bed with wooden frame and headboard. The bed frame and headboard were steamed 7 times during the study period, but small numbers of bed bugs were still found on the bed at 28 wk. Treatment III had one apartment without a bed. The apartment was clean, was sparsely furnished, and had little clutter. However, bed bug counts never became zero in more than two consecutive visits. The resident slept on one of the sofas every day and the sofas were treated 12 times during the 28 wk period. He used household cleaners frequently trying to reduce bed bugs on the sofa in addition to our biweekly treatment. The elimination failure may be partly due to insecticide resistance in the bed bugs, which is prevalent, based on recent studies [12,15]. Treatment IV had one cluttered apartment. The bed and sofa areas were very difficult to inspect and treat. Two bed bugs were found during the 28 wk inspection. These cases demonstrate that, even with very careful inspections and treatments, a small portion of the apartments can still present challenges to control. The finding is consistent with that reported by Cooper et al. (2015b), where 3 of the 55 treated apartments required ≥13 visits to eliminate bed bug infestations in similar settings [27]. 

The study site had a high bed bug infestation rate (20%) at the beginning of the study. Ineffective bed bug management practices was one of the primary reasons for the high infestation levels. In our study, 100% of the low-level infestations were eliminated using non-chemical methods alone, and the majority of the more severely infested units were eliminated with reduced chemical usage combined with non-chemical methods, demonstrating the effectiveness of a threshold-based approach. Among apartments with higher numbers (≥11) of bed bugs, treatment III achieved 89% (8 out of 9) elimination. The amount of liquid residual insecticide used per apartment in treatment III was also much less compared to chemical control studies conducted in similar environments [9,37,38], even though those studies lasted shorter periods than that in this study and eliminated ≤86% of the infestations. For future application of a threshold-based treatment approach, the threshold should be adjusted based on housing type, number of beds in each apartment, or the sampling method to be used. If more interceptors are placed for monitoring bed bugs over a longer period, the threshold can be adjusted to a higher number and vice versa. Additional interceptors should lessen the amount of intervention required for eliminating very low-level bed bug populations. For instance, in a recent study an average 28 interceptors were placed in each one bedroom apartment for 2 wk to determine the presence of bed bugs [25]. Using this sampling method, 94% (63 out of 67) of the infested apartments with ≤10 bed bugs were eliminated after 40 wk simply by mass trapping (mean number of 29 interceptors per apartment) and installing encasements to the mattresses and box spring.

## 5. Conclusions

A threshold-based treatment approach, based upon bed bug infestation level, can eliminate infestations while avoiding or minimizing insecticide use. Low-level bed bug infested one-bedroom apartments (≤12 bed bugs based on interceptor count and visual inspection) were eliminated rapidly with non-chemical methods alone. Higher level bed bug infestations required more number of visits to be eliminated. Combination of non-chemical and chemical treatment used significantly less liquid residual insecticide spray compared to chemical control. Comprehensive pre-treatment preparation of apartments is not necessary for eliminating most of the bed bug infestations. Applying these findings in future bed bug management will help save money and eliminate bed bugs rapidly and safely.

## Figures and Tables

**Table 1:** Initial bed bug counts based on combination of visual inspection and interceptors.

Treatment	n	Bed Bug Count		
		Median	Range	Mean ± SEM *
**I—Non-chemical only**	8	4	1–12	5 ± 2a
**II—Chemical control only**	9	4	1–12	5 ± 1a
**III—Non-chemical and chemical**	7	21	14–208	51 ± 27b
**IV—Chemical control only**	8	46	11–96	47 ± 11b

* Means within the same column followed by the same letters are not significantly different (Tukey’s HSD test, *p* > 0.05).

**Table 2:** Treatment information of the infested apartments.

Treatment	Mean Dust Usage Per Apartment (g) *	Mean Spray Usage Per Apartment (Liter) *	Mean (± SEM) Initial Treatment Time Per Apartment (Min) *	Median (Min, Max) Number of Re-Treatments before Bed Bugs Were Eliminated ^2^	Median (min, max) Number of Biweekly Visits Per Apartment before Bed Bugs were Eliminated ^2^
I	NA ^1^	NA	57 ± 8 a	0 (0–1)	1 (1–4)
II	6 ± 2a	1.0 ± 0.3 ab	87 ± 8 a	1 (0–11)	3 (1–10)
III	6 ± 1a	0.8 ± 0.2 a	97 ± 21 a	2 (0–8)	6 (1–9)
IV	10 ± 3a	1.9 ± 0.4 b	98 ± 22 a	6 (0–10)	7 (1–12)

^1^ Not applicable; ^2^ One apartment in each treatment was excluded from the analysis. They were either terminated early or elimination was not achieved or confirmed at the end of the study; * Means within the same column followed by the same letters are not significantly different (Tukey’s HSD test, *p* > 0.05).
